# Role of Orbicularis Oculi Resection in External Levator Advancement for Aponeurotic Blepharoptosis: A Prospective Randomised Controlled Trial

**DOI:** 10.1007/s00266-026-05617-5

**Published:** 2026-01-23

**Authors:** Sira Rojanasakul, Bunyada Putthirangsiwong

**Affiliations:** 1 Department of Ophthalmology, Faculty of Medicine Ramathibodi Hospital, Mahidol University, 270 Rama VI Road, Ratchathewi, Bangkok, 10400 Thailand; 2 Department of Ophthalmology, Chaophraya Abhaibhubejhr Hospital, 32/7, Prachin Anusorn Road, Mueang Prachin Buri, Prachin Buri 25000 Thailand

**Keywords:** Orbicularis oculi resection, External levator advancement, Aponeurotic ptosis

## Abstract

**Background:**

Removal of the skin and preseptal orbicularis oculi is the initial step in upper eyelid surgery. Preseptal orbicularis oculi removal has been strongly associated with dry eye symptoms due to sluggish eyelid closure and lagophthalmos. We aimed to investigate the effects of concurrent upper blepharoplasty and external levator advancement (ELA) surgery with or without orbicularis oculi resection on dry eye syndrome and eyelid morphology in Southeast Asian populations.

**Methods:**

This prospective, single-centre, double-blind, randomised controlled trial involved 20 Thai patients (40 eyes) with aponeurotic blepharoptosis and excess eyelid skin undergoing combined upper blepharoplasty and ELA surgery. Patients were randomised into a skin–muscle excision group (group A) or a skin-only excision group (group B). Dry eye parameters including tear break-up time, Oxford ocular surface staining, Ocular Surface Disease Index, eyelid appearance, and patient satisfaction were evaluated preoperatively and on postoperative days 7, 30, and 90.

**Results:**

Preseptal orbicularis oculi excision had no statistically significant impact on dry eye parameters, eyelid appearance, or patient satisfaction. For both groups, surgery increased the marginal reflex distance 1 without causing significant lagophthalmos, indicating successful ptosis correction irrespective of muscle excision. There were no discernible differences in postoperative appearance between the two groups, and the patients reported high satisfaction with their treatment.

**Conclusions:**

Combined upper blepharoplasty and ELA surgery, with or without resection of the preseptal orbicularis oculi, may be a safe and potentially effective procedure for patients with aponeurotic blepharoptosis and excess eyelid skin. Our findings demonstrate no evidence of a difference in correlation between either of these techniques and postoperative dry eye parameters or eyelid appearance. Further studies with larger sample sizes and longer follow-up periods are warranted.

**Level of Evidence I:**

This journal requires that authors assign a level of evidence to each article. For a full description of these Evidence-Based Medicine ratings, please refer to the Table of Contents or the online Instructions to Authors www.springer.com/00266.

## Introduction

Dry eye syndrome (dry eye) is a multifactorial condition affecting the ocular surface. The two main subtypes of dry eye are aqueous tear deficiency and evaporative dry eye. Eyelid abnormalities are intrinsic etiological factors leading to increased evaporative loss that can contribute to tear film instability and subsequent dry eye [1].

Upper eyelid ptosis is classified by onset (congenital or acquired) and further subclassified by cause (neurogenic, aponeurotic, myogenic, mechanical, or traumatic). The most common type of acquired upper eyelid ptosis is aponeurotic, which is caused by aponeurotic attenuation or dehiscence of the levator aponeurosis from the superior border of the tarsus. Surgical correction involving reattachment of the levator aponeurosis to its insertion is the mainstay of treatment. Surgical management can be performed using an internal/posterior approach (Müller muscle–conjunctiva resection, posterior approach white-line levator advancement) or external/anterior approach (external levator advancement [ELA]), the choice of which mostly depends on the surgeon’s preference. However, an external approach is preferred in circumstances including severe ocular surface diseases, cicatricial conjunctival diseases, and cases involving poor levator function [2]. Furthermore, concomitant upper blepharoplasty and ELA can be considered when patients have both ptosis and dermatochalasis.

Excision of excess skin with or without the underlying orbicularis oculi is the initial step of combined upper blepharoplasty and ELA surgery. Excision of the preseptal orbicularis oculi has been reported to improve the surgical technique by facilitating the exposure of deep structures, debulking upper eyelid fullness, forming a crease, and reducing the load on the levator [3–7]. However, various studies have recommended skin-only excision because it results in lower incidences of postoperative pain, bruising, eyelid oedema, and, in particular, dry eye [8–10]. The proposed mechanisms of dry eye include disrupted blinking leading to sluggish eyelid closure [9]. Additionally, excision of the preseptal orbicularis oculi leads to eyelid hollowness for some patients and increases the risk of marginal reflex distance 1 (MRD1) reduction following upper eyelid blepharoplasty [8, 11].

There is no clear evidence on whether it is better to excise or preserve the preseptal orbicularis oculi during combined upper blepharoplasty and ELA surgery. Data on patients of Asian ethnicity are also lacking. The purpose of this study is to compare the impacts of concurrent upper blepharoplasty and ELA with and without preseptal orbicularis oculi excision on postoperative outcomes including dry eye parameters, eyelid appearance, and patient satisfaction.

## Methods

### Patients

This prospective, single-centre, double-blind, randomised controlled trial was conducted at Ramathibodi Hospital in Bangkok, Thailand. Patients diagnosed with aponeurotic ptosis between July 2022 and February 2023 were screened for study entry. Patients were included if they were over the age of 18, had good levator function (levator excursion > 12 mm), and agreed to participate in the study. Exclusion criteria included a history of ptosis from other causes; prior dry eye or ocular surface disease; usage of eye drops or other medications potentially causing dry eye; previous eyelid or eyebrow surgery; previous periocular trauma; and an MRD1 difference between the eyes exceeding 2 mm. This study adhered to the principles of the Declaration of Helsinki and received approval from the Institutional Review Board of the Human Research Ethics Committee at Ramathibodi Hospital, Mahidol University, Bangkok, Thailand (IRB number: COA. MURA2022/318). Informed consent was obtained from patients who met the inclusion criteria.

Patients were randomly assigned to group A (concomitant skin–orbicularis oculi excision blepharoplasty and ELA) or group B (concomitant skin-only excision blepharoplasty and ELA) through block randomisation using a web-based software [12]. Each patient received a sealed opaque envelope containing their group allocation. All patients and the investigator (S.R.) were fully blinded to the group allocation until the completion of all data collection. The surgeon (B.P.), being unblinded to the study’s allocation, knew which operation to perform on the patients on the day of surgery.

The primary outcome measure was postoperative dry eye following combined upper blepharoplasty and ELA surgery. This was objectively assessed by measuring the tear break-up time (TBUT) [13] at day 90. The secondary outcome measures were other dry eye parameters, including the Oxford Ocular Surface Staining (OOSS) scale [14]; the Thai version of the Ocular Surface Disease Index (OSDI), calculated from a 12-item questionnaire [15]; postoperative MRD1; degree of postoperative lagophthalmos; eyelid aesthetics, assessed using the Strasser grading system and Global Aesthetic Improvement Scale (GAIS) [16]; and patient satisfaction, assessed using the Thai version of the Client Satisfaction Questionnaire (CSQ-8) [17]. All data were collected by a single investigator (S.R.) preoperatively and on postoperative days 7, 30, and 90, with patients completing the CSQ-8 on the final visit.

Preoperative examination included a thorough assessment of eyelids, eyelashes, and anterior segments, with close attention paid to any signs of preexisting dry eye or meibomian gland dysfunction. To determine MRD1, the corneal light reflex was induced by illumination, and the distance in millimetres from the central upper eyelid margin to the corneal light reflex was then measured. Patients were asked to gently close their eyes, and any degree of lagophthalmos was measured as the distance in millimetres between the upper and lower eyelid margins. In a dark, air-conditioned room without direct air currents, TBUT was assessed using fluorescein-impregnated strips, following the steps recommended by the Tear Film Ocular Surface Society. TBUT was measured three times for each eye, and the mean value was recorded and later calculated for analysis. After TBUT measurement, OOSS was graded by comparing the density of corneal and conjunctival fluorescein staining with that in panels A–E and > E of a supplied reference chart (grades 0–IV and V, respectively) [14], which quantifies the severity of epithelial surface damage caused by dry eye.

The OSDI consists of 12 questions designed to subjectively evaluate symptoms of dry eye. OSDI scores, which can range from 0 to 100 (0–12, normal; 13–22, mild dry eye; 23–32, moderate dry eye; > 33, severe dry eye), were calculated using the patients’ questionnaire responses. Preoperative and 90-day postoperative standardized photographs of the patients’ eyes, open and closed in primary position, were taken for aesthetic assessment by three independent, blinded assessors, including one general ophthalmologist, one oculoplastic surgeon, and one plastic surgeon. The aesthetic outcome was evaluated using the Strasser grading system, which assigns scores of 0 to 3 (0, perfect; 1, noticeable; 2, obvious; 3, obvious and deforming) to the severity of flaws in each of five categories: asymmetry, contour deformity, scar, distortion, and malposition. The total category scores are rated as good (0–4 points), mediocre (5–14 points), or poor (15 points). Additionally, the GAIS was used to assess overall postoperative outcomes (Table 1). The scores of GAIS are rated as improved (1–3) and not improved (4–5) postoperative appearance. Follow-up examinations on postoperative days 7, 30, and 90 followed the same processes, with the addition of the CSQ-8 questionnaire on the final visit. Total CSQ-8 scores may range from 8 to 32, with higher scores indicating greater satisfaction.

**Table 1:** Global aesthetic improvement scale

Rating	Description
1. Exceptional improvement	Excellent corrective result
2. Very improved patient	Marked improvement of the appearance, but not completely optimal
3. Improved patient	Improvement of the appearance, better compared with the initial condition, but a touch-up is advised
4. Unaltered patient	The appearance substantially remains the same compared with the original condition
5. Worsened patient	The appearance has worsened compared with the original condition

The required sample size was calculated using TBUT data from a previous study [18], using a comparing two independent population means (*α* = 0.05, power = 0.8, pooled standard deviation = 2.0660). A sample size of 20 eyes (10 patients) was determined for each group to sufficiently detect a 2-second difference in TBUT between Group A and Group B. All the measurements were performed for each individual eye rather than by averaging both eyes in one participant. Baseline characteristics of all patients were recorded and analysed. Data normality was assessed using the Kolmogorov–Smirnov and Shapiro–Wilk tests. Continuous variables were expressed as mean ±standard deviation (SD) or median (IQR), as appropriate. Categorical variables were summarized as frequencies and percentages. Comparisons between categorical variables were performed using the Chi-square test or Fisher’s exact test, as appropriate, while continuous variables were compared using the Student’s t test or Mann–Whitney U test. Repeated measurements of TBUT, OOSS, OSDI, MRD1, and lagophthalmos at each visit were analysed using a linear mixed-effects model. To account for correlations between repeated measurements from both eyes of the same participant, the subject was included as a random effect, while laterality was modelled as a fixed effect to adjust for potential inter-eye differences. Data analysis was performed using STATA version 18.0, with p values <0.05 considered statistically significant.

### Surgical Procedures

All patients underwent bilateral upper blepharoplasty with ELA as a day case surgery performed under local anaesthesia and by a single surgeon (B.P.) using the same techniques for all patients. The skin was marked in a standard fashion by pinching with forceps to estimate the excess skin, an anaesthetic preparation (20 mg/mL lidocaine hydrochloride [Xylocaine] and 12.5 μg/mL adrenaline) was injected along the surgical marking on the upper eyelid, and a skin incision was made along the skin marking. For patients in group A, the skin and underlying preseptal orbicularis were excised to the extent of the excess skin removal using the pinching forceps techniques, and for patients in group B, only the skin was removed. Subsequently, the pretarsal orbicularis was incised along the upper eyelid margin to expose the upper border of the tarsus, which was dissected until the tarsal plate was adequately exposed. The orbital septum was then incised, and the preaponeurotic fat pads including the medial and lateral lobes were identified and debulked. Following this, the distal part of levator aponeurosis was identified and sutured to the 1/3 upper border of the tarsus with vicryl 6-0 sutures in an interrupted fashion, using a three-stitch adjustment to achieve the proper eyelid height and contour. The lid crease was further adjusted by suturing the pretarsal orbicularis to the aponeurosis of the levator muscle with vicryl 6-0 sutures in an interrupted fashion. Any bleeding encountered during the procedure was stopped using bipolar cautery. Finally, the skin was closed continuously using nylon 6-0 sutures. After the operation, patients were discharged with oral analgesics and topical antibiotics ointment to apply to the surgical wound twice daily. Sutures were removed during the first follow-up visit on postoperative day 7, and the examinations mentioned above were conducted by one investigator (S.R.). The study protocol permitted patients with significant dry eye symptoms to receive treatment with artificial tears. However, no patients required such treatment in this study.

## Results

A total of 22 patients (44 eyes) were enrolled in the study, with 11 patients (22 eyes) allocated to Group A and 11 patients (22 eyes) to Group B. One patient in group A was lost to follow-up, and one patient in group B withdrew from the study after developing severe dry eye unrelated to the intervention, resulting in 10 patients (20 eyes) in each group available for analysis. Of these, 15 patients were women and 5 were men, with a mean age of 64.1 years in group A and 68.1 years in group B. Baseline characteristics were comparable between the two groups (Table 2).

**Table 2:** Baseline characteristics of patients with aponeurotic ptosis

Characteristic	Group A	Group B	*P* value
*Gender*, *n* (%)			
Male	3 (30)	2 (20)	1.000
Female	7 (70)	8 (80)	
Age-yr, mean (SD)	64.1 (15.4)	68.1 (9.7)	0.496
TBUT-sec, median (IQR)	4.4 (3.55–6.29)	4.3 (3.42–4.63)	0.992
*Oxford staining-grade, n (%)*			
Grade 0	8 (80)	10 (100)	0.474
Grade 1	2 (20)	0 (0)	
OSDI-score, median (IQR)	15.64 (4.17–27.08)	4.17 (0.0–10.42)	0.127
MRD1-mm, median (IQR)	1.375 (0.5–2.25)	1.375 (1.25–2.0)	1.000
Lagophthalmos-mm, median (IQR)	0	0	1.000

*P* values were derived from Fisher’s exact test where expected cell counts were < 5; otherwise, Pearson’s chi-square test was used

Compared with patients in group B, those in group A had a longer TBUT on postoperative day 7, but values of TBUT in group A on postoperative days 30 and 90 were slightly shorter than the respective values in group B. However, the differences in TBUT between the two groups were not statistically significant. Moreover, patients in group A had slightly greater OOSS than patients in group B on postoperative days 7, 30, and 90, but the differences between groups were also not statistically significant. Additionally, patients in group A experienced mild dry eye symptoms, whereas those in group B reported no dry eye symptoms, as shown by the values of the OSDI. Nevertheless, there were no significant differences in OSDI between the groups on any of the follow-up days. Up to 0.2 mm in lagophthalmos was detected in both groups on postoperative day 7. However, the measurement finally returned to baseline by the final visit. The data are shown in Table 3.

**Table 3:** Postoperative dry eye parameters and differences between groups

Parameters	Group A	Group B	Difference (95% CI)	*P* value
*TBUT (seconds), mean (SE)*				
Day 7	8.28 (0.78)	6.25 (0.78)	1.46 (− 0.72–3.64)	0.190
Day 30	6.33 (0.87)	6.78 (0.86)	− 1.02 (− 3.44–1.41)	0.411
Day 90	6.54 (1.28)	6.82 (1.27)	− 0.84 (− 4.40–2.72)	0.643
*OOSS (grade), mean (SE)*				
Day 7	0.71 (0.19)	0.45 (0.19)	0.061 (− 0.46–0.58)	0.818
Day 30	0.45 (0.21)	0.34 (0.21)	−0.10 (− 0.69–0.48)	0.732
Day 90	0.61 (0.33)	0.34 (0.33)	0.064 (− 0.85–0.98)	0.891
*OSDI (score), mean (SE)*				
Day 7	11.91 (2.29)	4.54 (2.29)	6.77 (− 0.75–14.29)	0.077
Day 30	16.60 (2.47)	8.04 (2.45)	7.95 (0.003–15.90)	0.050
Day 90	13.25 (3.42)	2.63 (3.38)	10.02 (− 0.252–20.29)	0.056
*Lagophthalmos (mm), mean (SE)*				
Day 7	0.15 (0.05)	0.10 (0.05)	0.05 (− 0.087–0.19)	0.474
Day 30	0 (0.05)	0.12 (0.05)	− 0.13 (− 0.27–0.017)	0.084
Day 90	0 (0.05)	0 (0.05)	0.00 (− 0.14–0.14)	0.993

**Fig. 1:**
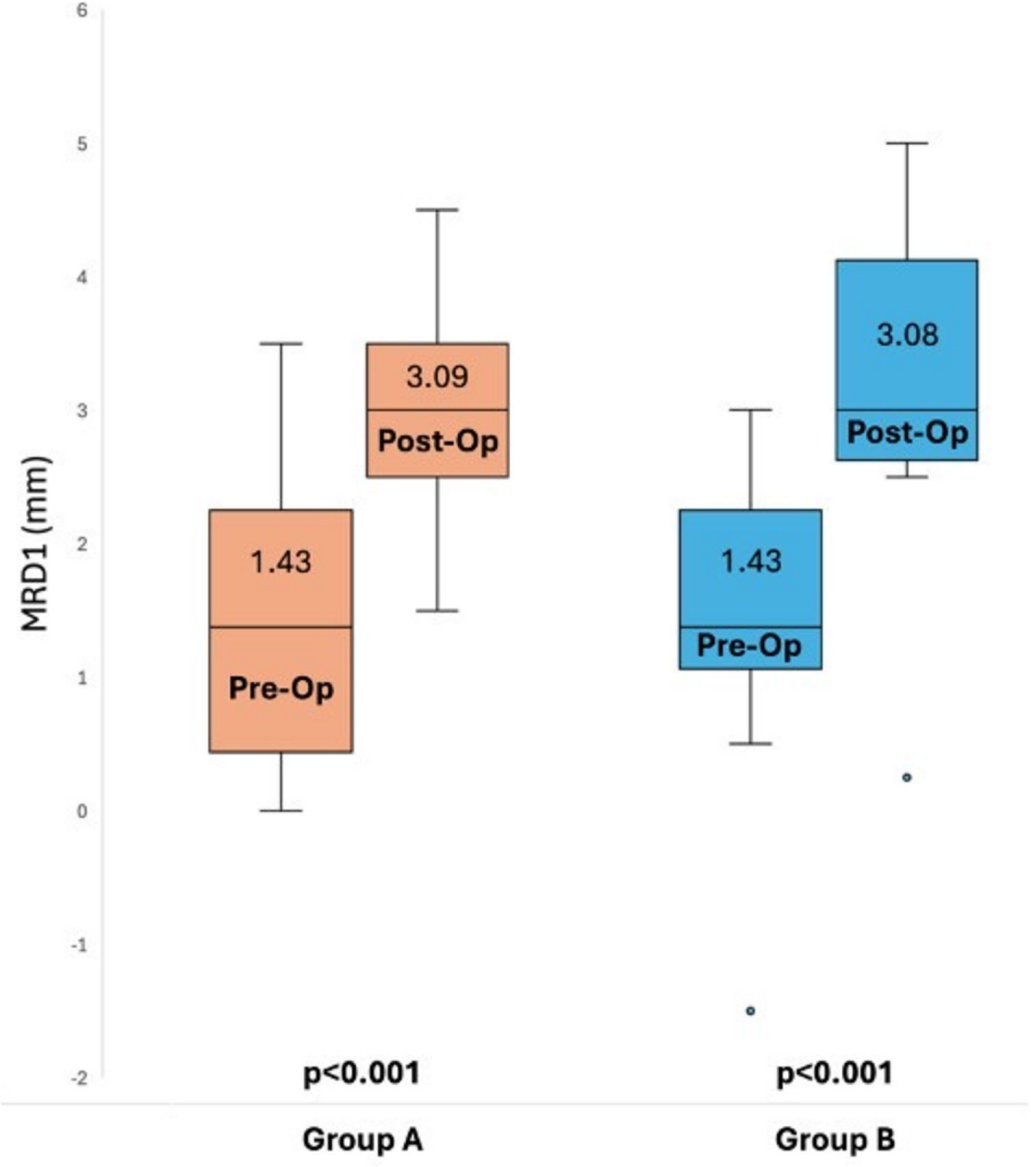
Box plots of pre-and postoperative values for marginal reflex distance 1 (mm), (orange) the skin–orbicularis oculi excision group and (blue) skin-only excision group

For all patients, postoperative MRD1 was significantly greater than preoperative MRD1, and there was no significant difference in this MRD1 increase between the groups (*p* = 0.95). The average increase in MRD1 was 1.66 (95% CI, 1.06–2.27; *p* < 0.001) in group A and 1.65 (95% CI, 1.06–2.26; *p* < 0.001) in group B (Fig. 1).

**Fig. 2:**
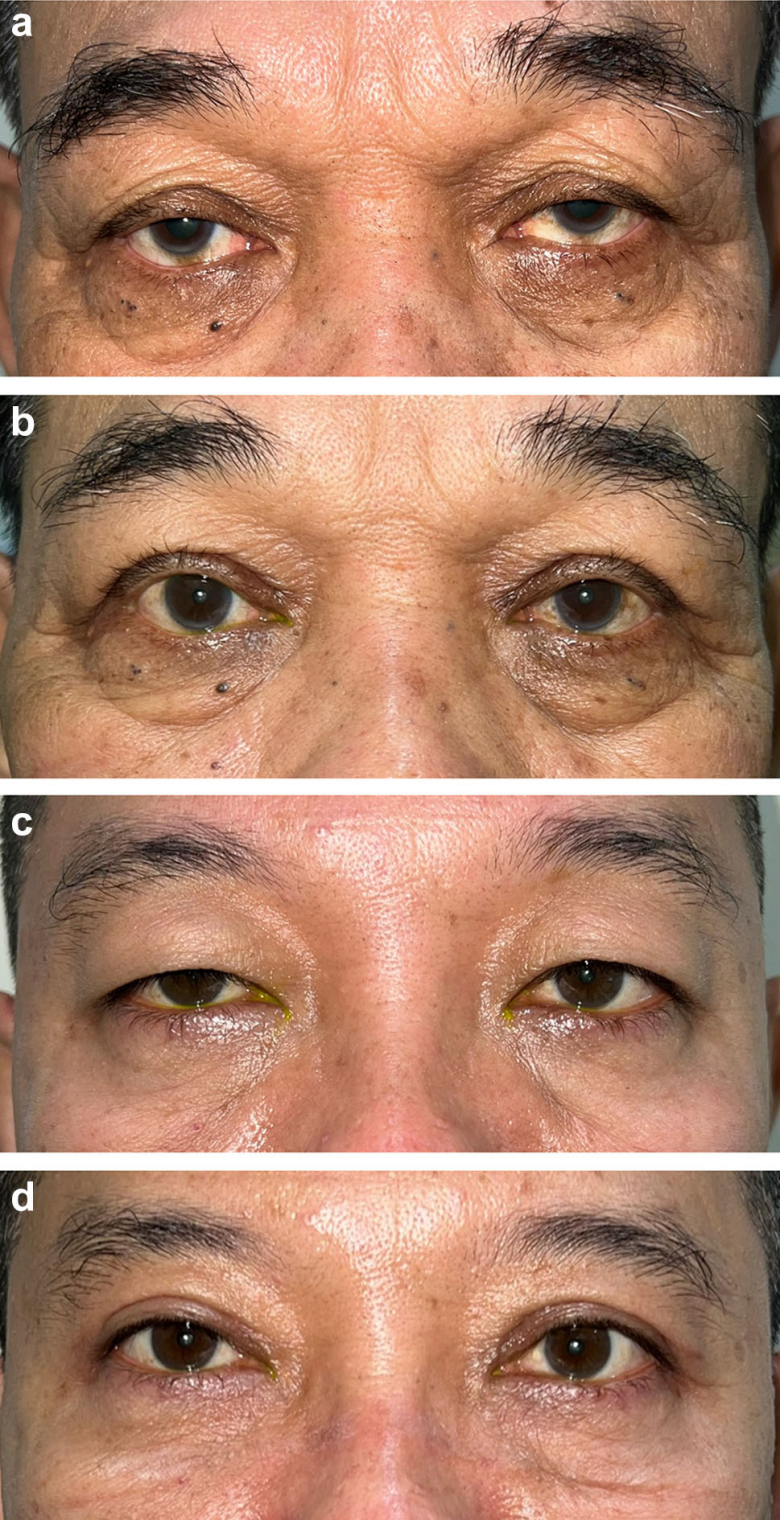
Preoperative (**a**, **c**) and postoperative (**b**, **d**) photographs of two patients in a skin–muscle excision group (**a**, **b**) and in a skin-only excision group (**c**, **d**)

The three independent, blinded assessors gave similar scores using both the Strasser grading system and the GAIS. Using the Strasser grading system, the average scores for asymmetry, contour deformity, scarring, distortion, and malposition were 0.43, 0.23, 0.43, 0.43, and 0.43, respectively, in group A and 0.23, 0.30, 0.44, 0.30, and 0.33, respectively, in group B (Table 4). The average GAIS score was 2.1 in group A and 2.33 in group B (*p* = 0.578), indicating improved postoperative appearance. There were no statistically significant differences in any of the above scores between the two groups (Fig. 2).

**Table 4:** Aesthetic outcomes using strasser grading system

	Asymmetry	Contour deformity	Scar	Distortion	Malposition
Group A	0.43	0.23	0.43	0.43	0.43
Group B	0.23	0.30	0.44	0.30	0.33
Difference (95% CI)	0.07 (−0.27, 0.41)	− 0.07 (− 0.37, 0.23)	− 0.33 (− 0.69, 0.33)	0.13 (− 0.21, 0.47)	0.1 (− 0.22, 0.42)
P value	0.700	0.663	0.069	0.441	0.541

The mean CSQ-8 score (± standard error of the mean) was 29.33 ± 1.94 in group A and 30.67 ± 2.06 in group B (*p* = 0.177), indicating that all patients were highly satisfied with the postoperative outcomes.

## Discussion

In the current study, the postoperative dry eye parameters (TBUT, OOSS, and OSDI) indicated no statistically significant differences in dry eye problems between patients undergoing skin–orbicularis oculi excision blepharoplasty with ELA (group A) and patients undergoing skin-only blepharoplasty with ELA (group B). The increased TBUT measured on postoperative day 7 in group A compared with group B could be explained by a mechanism involving increased tear production resulting from sluggish eyelid closure and lagophthalmos related to orbicularis oculi removal. However, the TBUT values of group A during subsequent follow-ups were lower than those of group B. Since none of the above differences in dry eye parameters were statistically significant, the resection of orbicularis oculi during blepharoplasty with ELA may not be a significant factor affecting postoperative dry eye. Kiang et al. [9] and Mostafa [18] reported a higher rate of postoperative dry eye problems in patients who had undergone orbicularis oculi removal during upper blepharoplasty compared with those who had not. In contrast, Maria et al. [19] found that resection of the orbicularis oculi during blepharoplasty had no impact on long-term dry eye symptoms and signs. They further proposed that the removal of the excess eyelid tissue leads to improved eyelid function, subsequently resulting in a reduction of dry eye symptoms and signs. Our study, which focused on short-term follow-up, found that postoperative dry eye parameters are not statistically affected by resection of the orbicularis oculi during combined upper blepharoplasty with ELA surgery. According to the findings of Maria et al. [19] and our current study, orbicularis oculi resection during blepharoplasty with ELA does not result in either short-term or long-term dry eye complications.

Additionally, our study demonstrated a statistically and clinically significant postoperative improvement in MRD1 in both groups. These findings indicate that successful aponeurotic ptosis correction can be achieved by ELA regardless of whether the orbicularis oculi is resected during blepharoplasty. Our results contradict previous findings that resection of the orbicularis oculi significantly increases the risk of postoperative MRD1 reduction [11, 20, 21]. Brown and Putterman [20] compared the change in MRD1 resulting from Müller-muscle conjunctival resection alone with that resulting from Müller-muscle conjunctival resection combined with skin–orbicularis oculi–fat excision blepharoplasty and found that the latter procedure reduced the postoperative eyelid elevation. Moore et al. found no difference in MRD1 change between Müller-muscle conjunctival resection alone and Müller-muscle conjunctival resection combined with skin-only excision blepharoplasty. These findings indirectly demonstrate a relationship between orbicularis oculi resection during blepharoplasty and postoperative MRD1 reduction. In support of this relationship, a more recent study found that orbicularis oculi removal is a significant surgical factor affecting postoperative MRD1 reduction [11]. In contrast, the current study found no significant difference in MRD1 change between skin–muscular flap excision and skin-only excision procedures. We postulate that intraoperative MRD1 adjustment during ELA surgery has a more important influence on postoperative outcomes than orbicularis oculi resection during blepharoplasty.

In the existing literature, the role of orbicularis oculi resection during blepharoplasty on aesthetic outcomes remains debatable. Some authors have advocated for the preservation of the muscle because it has no impact on the postoperative cosmetic outcomes but risks iatrogenic damage to the levator aponeurosis and a hollowed appearance [10]. Other authors have argued that excision of the orbicularis oculi can help to correct lateral hooding from the hypertrophic orbicularis oculi, resulting in cosmetic improvements [22]. Furthermore, as Asian people generally have prominent subcutaneous and retro-orbicularis fat on their upper eyelids, we initially proposed that orbicularis oculi removal may alleviate upper eyelid lumpiness and result in more cosmetically appealing outcomes. However, our current study found no statistically significant evidence to support this. Both the Strasser grading and GAIS scores revealed good overall postoperative outcomes, and the high CSQ-8 scores indicated that all patients were satisfied with the postoperative results. We therefore suggest that orbicularis oculi resection during upper blepharoplasty may not be an important factor affecting aesthetic outcomes and that this muscle should be conservatively excised only to provide convenient access to the levator aponeurosis during ELA surgery.

This study had a few limitations. The small sample size might have insufficient power to reveal significant differences in outcomes, potentially leading to a false negative. The authors also conducted the Bayesian Multilevel Regression [23] as a sensitivity analysis to provide more robust estimates given the small sample size. This analysis found results consistent in direction and magnitude across all parameters with those of the Mixed-effects Multilevel Regression used for our data analysis. In addition, our follow-up periods were limited to 90 days, whereas more accurate outcomes could be observed at least 6 months postoperatively. It is possible that dry eye problems had not manifested at the time of observation or may have resolved during a subsequent period of time. Finally, the amount of the orbicularis oculi resected from each patient in group A was not quantified and evaluated. Variable amount of muscle removed could impact the postoperative parameters and outcomes. Further research with a larger sample size, longer follow-up periods and quantified amount of orbicularis oculi resection may be necessary to obtain more accurate data that are applicable to real clinical outcomes.

We found that upper blepharoplasty combined with ELA surgery shows promise as an effective procedure with no evidence of significant postoperative complications, regardless of whether the orbicularis oculi is concurrently resected. We therefore conclude that resection of the orbicularis oculi may not significantly influence postoperative dry eye or aesthetic outcomes, and we recommend routinely preserving this muscle during upper blepharoplasty to maintain its normal morphology and physiology. However, in circumstances which orbital septum incision is considered (e.g. surgical debulking of preaponeurotic fat pads, ELA), modest amounts of orbicularis oculi can be acceptably excised to improve exposure of the orbital septum without unfavourable sequelae.
